# Supplementary material for: Saccharomyces boulardii (CNCM I-745) improves intestinal damage in sepsis by remodeling intestinal flora structure
Source: Front Cell Infect Microbiol. 2025 Jul 31;15:1602792. doi: 10.3389/fcimb.2025.1602792 (PMC12350329; doi:10.3389/fcimb.2025.1602792)
Supplement: Supplementary file 1 [file DataSheet1.zip › summary/summary/8_Advanced_analyse/2_PICRUSt2_result/KEGG/Group.DW4_vs_DW0_vs_CW4_vs_CW0_vs_BW4_vs_BW0_vs_AW4_vs_AW0/PICRUSt2_KO_Group.DW4_vs_DW0_vs_CW4_vs_CW0_vs_BW4_vs_BW0_vs_AW4_vs_AW0_diff.pdf]

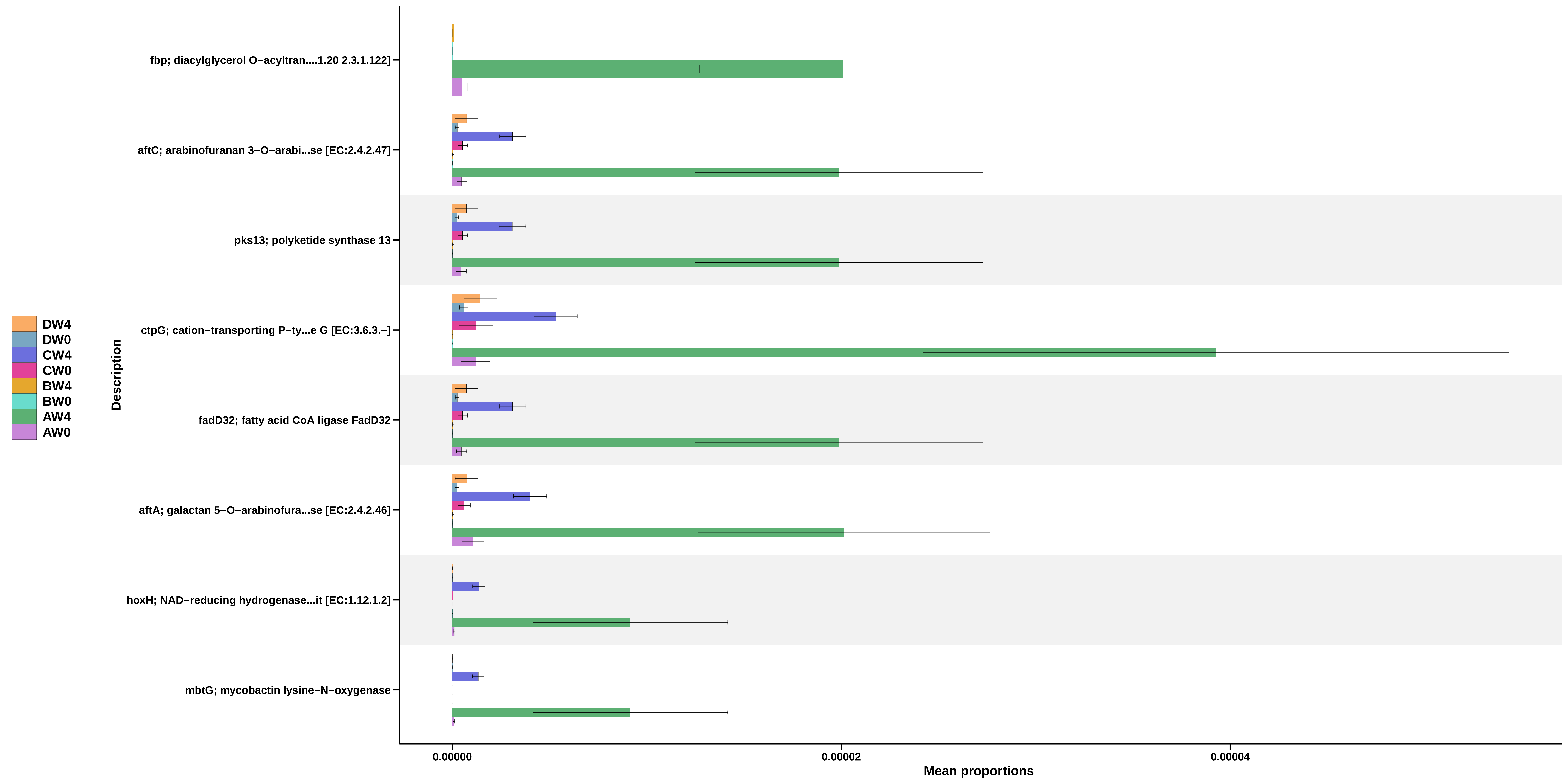

fbp; diacylglycerol O−acyltran....1.20 2.3.1.122]
aftC; arabinofuranan 3−O−arabi...se [EC:2.4.2.47]
pks13; polyketide synthase 13
ctpG; cation−transporting P−ty...e G [EC:3.6.3.−]
fadD32; fatty acid CoA ligase FadD32
aftA; galactan 5−O−arabinofura...se [EC:2.4.2.46]
hoxH; NAD−reducing hydrogenase...it [EC:1.12.1.2]
mbtG; mycobactin lysine−N−oxygenase
DW4
DW0
CW4
CW0
BW4
BW0
AW4
AW0
Description
0.00000
0.00002
0.00004
Mean proportions
